# Delivery of large transgene cassettes by foamy virus vector

**DOI:** 10.1038/s41598-017-08312-3

**Published:** 2017-08-14

**Authors:** Nathan Paul Sweeney, Jinhong Meng, Hayley Patterson, Jennifer E. Morgan, Myra McClure

**Affiliations:** 1 Jefferiss Research Trust laboratories, Imperial College London, London, United Kingdom; 2 The Dubowitz Neuromuscular Centre, Molecular Neurosciences Section, Developmental Neurosciences Programme, UCL Great Ormond Street Institute of Child Health, London, United Kingdom

## Abstract

Viral vectors are effective tools in gene therapy, but their limited packaging capacity can be restrictive. Larger clinically-relevant vectors are needed. Foamy viruses have the largest genomes among mammalian retroviruses and their vectors have shown potential for gene therapy in preclinical studies. However, the effect of vector genome size on titre has not been determined. We inserted increasing lengths of the dystrophin open reading frame in a foamy virus vector and quantified packaged vector RNA and integrated DNA. For both measures, a semi-logarithmic reduction in titre was observed as genome size increased. Concentrated titres were reduced 100-fold to approximately 10^6^ transducing units per ml when vector genomes harboured a 12 kb insert, approximately twice that reported for lentivirus vectors in a comparable study. This potential was applied by optimising foamy virus vectors carrying the full-length dystrophin open-reading frame for transduction of human muscle derived cells. Full-length dystrophin protein was expressed and transduced cells remained able to form myotubes *in vitro*. Foamy virus vectors are well-suited for stable delivery of large transgene cassettes and warrant further investigation for development as a therapy for Duchenne or Becker muscular dystrophy.

## Introduction

Two gene therapies, Glybera^1^ and Strimvelis^2^, have now been licensed in Europe for the treatment for rare genetic diseases and a number of clinical trials are showing promise for a range of diseases^3–6^. The advancement of gene engineering technologies^7^ and immunotherapies^8^ has broadened the spectrum of diseases that may be targeted by gene therapy. Viral vectors are often used to exploit the efficient mechanisms they have evolved to deliver and express their genomes. Their effectiveness is emphasised by the fact that both Glybera and Strimvelis use viral vectors (adenoassociated virus (AAV) and γ-retroviral vectors, respectively). However, as gene therapies become more complex and require the delivery of large or multiple transgenes, the packaging limits of viral vectors are increasingly restrictive.

The foamy viruses are a family of retroviruses from which self-inactivating clinically-relevant vectors have been developed^9–13^ and proven to be effective for gene therapy in large-animal models^14, 15^. Since foamy viruses have the largest mammalian retrovirus genomes, they are promising vectors for the delivery of large transgene cassettes^9^. However, the effect of transgene size on titre has not yet been determined. Uniquely, foamy virus reverse transcription can occur in the producer cell^16^, potentially allowing for efficient transduction of target cells with low dNTP availability. Furthermore, although foamy virus integration is mitosis-dependent^17^, virions can ‘wait’ for at least 30 days for cell division to occur by persisting at the centrosome^18^. These features enable efficient transduction of quiescent cells that will eventually divide, such as primary T cells and CD34+ cells^19^. Hence, an ability of foamy virus vectors to efficiently deliver large transgene cassettes could be of high value to gene therapy.

The muscular dystrophies are a group of diseases characterised by progressive weakening of muscles. The most common and severe type, Duchenne muscular dystrophy (DMD), affects approximately 1 in 3500 male births^20, 21^ and is caused by nonsense or frame-shift mutations in the dystrophin gene located on the X chromosome^22^. A closely related form, Becker muscular dystrophy (BMD), is also caused by mutations in the dystrophin gene but results in a milder phenotype since the reading frame is (generally) not disrupted, resulting in expression of partially functional truncated dystrophin^22^. Dystrophin is a large cytoskeletal protein responsible for anchoring the actin cytoskeleton to the extracellular matrix^23^. While different tissues express different isoforms, the full-length isoform, encoded by an 11 kb open reading frame (ORF), is predominantly expressed in skeletal muscle^22^. Gene therapy has the potential to correct or improve disease in DMD and BMD, but the size of the dystrophin ORF makes this challenging since it exceeds the packaging capacities of favoured viral vectors, such as AAV and lentivirus vectors^24, 25^. Hence, current DMD gene replacement strategies are limited to the delivery of truncated dystrophin ORFs known as micro- or mini-dystrophins^26^. Since full-length dystrophin expression cannot be restored by these strategies, successful therapy would at best result in a BMD-like phenotype. The delivery of the whole dystrophin ORF is a desirable approach, but clinically-relevant vectors able to deliver larger transgene cassettes efficiently are needed to achieve this.

This study aimed to define the effects of increasing the foamy virus vector (FVV) genome size on vector titre. As a clinically-relevant model, the potential for FVVs to deliver and express the full-length dystrophin ORF in human skeletal muscle-derived stem cells was evaluated.

## Results

### The effect of genome size on titre

**Figure 1:**
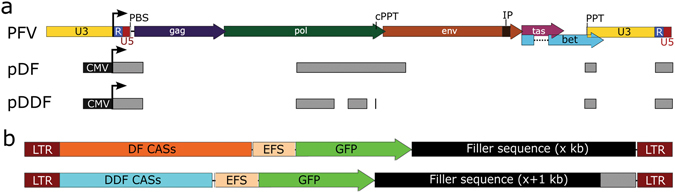
Vector designs to determine effect of size on titre. (**a**) Alignment of the PFV provirus sequence to the transfer plasmids pDF or pDDF. (**b**) Strategy to determine the effect of size on titre. Filler sequence added in increasing increments of 1 kb are controlled for by the difference in size between the CIS-acting sequences (CASs) of DF (top) and DDF (bottom). Arrows indicate the transcriptional start site for genomic RNAs. PBS - primer binding site. cPPT - central polypurine tract. IP - internal promoter. PPT - polypurine tract. CMV - cytomegalovirus promoter. LTR - long terminal repeat. EFS - elongation factor 1α short promoter. GFP - enhanced green fluorescent protein.

To determine the packaging capacity of FVVs, two vector backbones differing in size by approximately 1 kb were utilised. The first, DF (deleted foamy), has been described^9^, while the second, DDF (deleted deleted foamy), was constructed for this study by deleting sequences shown to be unnecessary in a parallel FVV system^11^. The sequence alignment maps for pDF and pDDF to the parent prototype foamy virus (PFV) genome is shown (Fig. 1a). Both backbones were tested in parallel to ensure that any effects on titre (from introducing 1 kb increments of filler sequence) were due to the increase in vector size rather than from the presence of novel sequences that could disrupt titre, as illustrated (Fig. 1b).

Using the dystrophin ORF (~11 kb) as a clinically-relevant sequence, we inserted increasing lengths of it (from 7 kb to full-length in 1 kb increments) behind the stop codon of a 1 kb EFS-GFP reporter cassette (Fig. 1b). The dystrophin sequences served as filler sequence and would not be translated from these vectors. Instead, additional vectors encoding the full-length dystrophin ORF under the control of the phosphoglycerate kinase (PGK) promoter were constructed. All vectors, the insert size and resulting provirus size in DF and DDF are shown in Table 1.

**Table 1:** FVV transfer plasmid inserts and the resulting provirus size.

Name	Insert size (bp)	Provirus DF (kb)	Provirus DDF (kb)
EFS-GFP	1009	4.55	3.48
EFS-GFP-dys7^a^	8001	11.55	10.46
EFS-GFP-dys8	9001	12.55	11.46
EFS-GFP-dys9	10000	13.55	12.46
EFS-GFP-dys10	11000	14.55	13.46
EFS-GFP-dysFL^b^	12059	15.61	14.52
PGK-GFP-WPRE^c^	1855	5.45	4.35
PGK-Dys	11568	15.16	14.06
PGK-Dys.GFP^d^	12297	15.89	14.78

^a^The numbers [-dys(n)] correspond to the length of 5′ dystrophin ORF in kb. ^b^FL indicates inclusion of the full-length ORF. ^c^WPRE - woodchuck hepatitis virus post-transcriptional regulatory element. ^d^Dys.GFP encodes a full-length dystrophin-GFP fusion protein.

The transfer plasmids shown in Table 1 were cotransfected with Gag, Pol and Env expression constructs into 293 T cells to produce FVV and determine their relative titres. Filtered cell-culture supernatant was concentrated 100-fold by ultracentrifugation and added to HT1080 fibroblasts, a cell type efficiently transduced by FVVs, to measure functional titre. However, since GFP expression was not detected from cells transduced with vectors containing any length of the dystrophin ORF, alternative methods for titration were employed.

**Figure 2:**
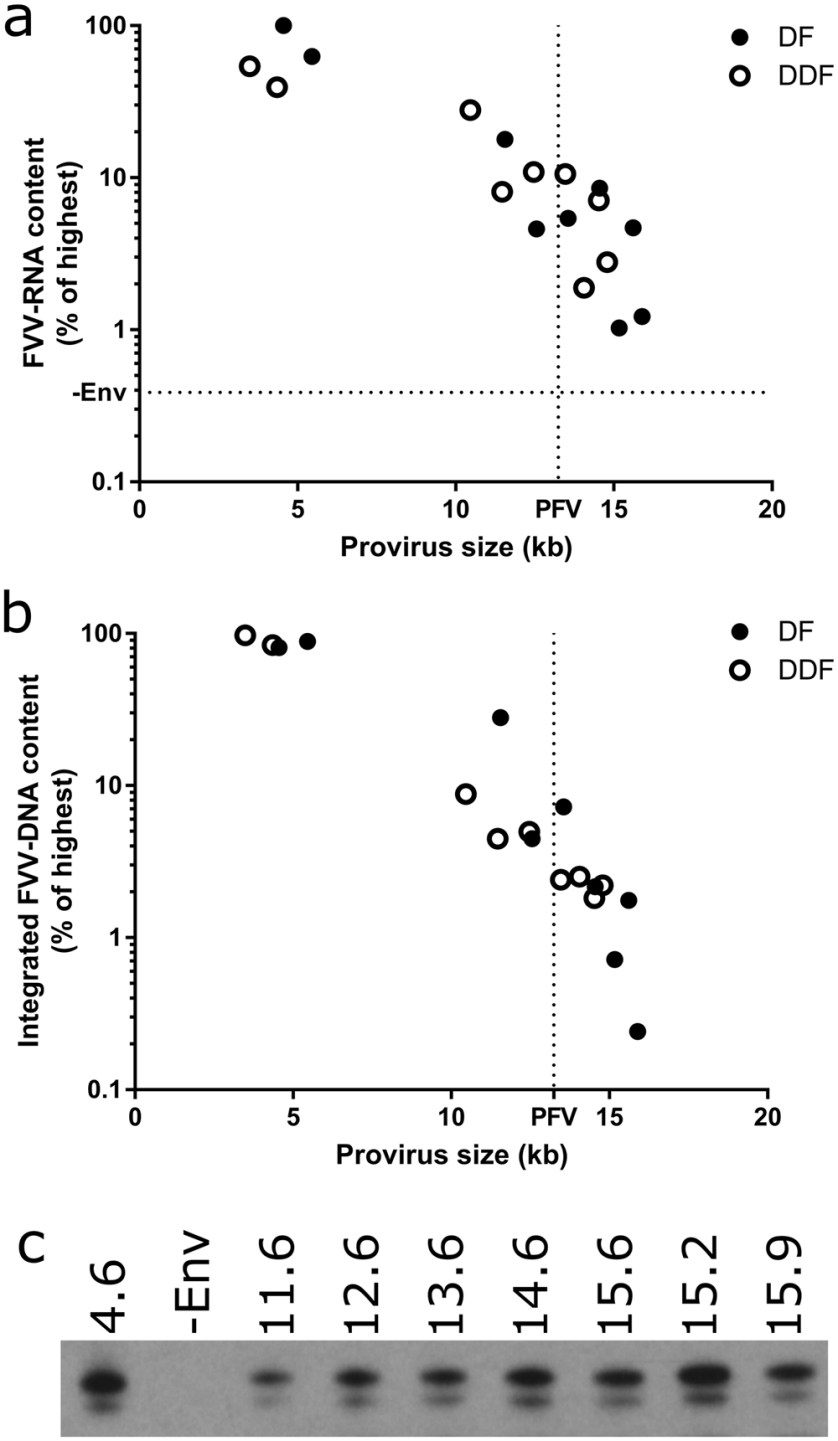
Effect of FVV size on titre. (**a**) The relative amounts of FVV-RNA in concentrated vector preparations was quantified by RT-qPCR and is shown as a percentage of the vector with the highest RNA content. The dashed horizontal line shows the relative amount of FVV-RNA in the -Env control. (**b**) Genomic DNA was extracted from HT1080 cells transduced by the FVVs and the relative amounts of integrated FVV-DNA was determined by Alu-qPCR. Results are presented as percent of the vector with the highest amount of integrated DNA. No integrated FVV-DNA was detected from the -Env control. For reference, the PFV provirus size is indicated by a dashed vertical line in (**a**,**b**). Data is representative of 2 independent experiments. (**c**) Equal volumes of vector-producing cell-culture supernatant was separated by SDS-PAGE and analysed by Western blot using human anti-PFV serum. The characteristic 71/68 kDa Gag doublet is shown in a representative blot. The vector (provirus) sizes are given in kb above the corresponding lane except for the negative control (indicated as -Env). Full length blot is shown Fig. S6a.

Viral RNA was extracted from concentrated FVV to determine the relative abundance of packaged FVV-RNA by RT-qPCR (Fig. 2a). Since FVV particle release is dependent on the presence of Env^27^, a control transfection including all components except for the Env-encoding plasmid (using pDF-EFS-GFP transfer plasmid) served as a control for unpackaged RNAs. In addition to measuring packaged FVV-RNA, the concentrated vectors were added to HT1080 cells. One passage post-transduction, genomic DNA was isolated and the relative amounts of integrated FVV-DNA was determined by Alu-qPCR (Fig. 2b). Unintegrated FVV-DNA and plasmid DNA is not quantified using this technique. Finally, the amount of FVV Gag released into the supernatant of vector-producing 293 T cells was compared by Western blot (Fig. 2c).

As shown in (Fig. 2a,b), the size of the vector genome is inversely related to titre when measured by either the FVV-RNA content (RT-qPCR) or the amount of FVV-DNA integrated into the genome of transduced cells (Alu-qPCR). Insertion of 7 kb of filler sequence caused the provirus size to increase approximately 3-fold (Table 1) but leads to an almost 10-fold reduction in titre. Titre continued to decrease as the vector size increased in a semi-logarithmic manner, with the largest vectors tested having a 100-fold lower titre than the small GFP-only vectors. Vectors produced using different transfer vectors, DF or DDF, had similar titres at all sizes tested, excluding the presence of prominent detrimental elements in the filler sequences. Although an equal mass of transfer vector was used for each transfection, meaning the transfected plasmid copy number decreased as its size increased, this was not a major factor in titre reductions, since maintaining a constant molar ratio of transfected plasmids resulted in similar levels of released FVV-RNA (Fig. S1).

In contrast to the inverse correlation between FVV-RNA and integrated FVV-DNA quantities with transfer vector size, the amount of Gag released into the transfected cell-supernatant was similar for all sizes of vectors tested (Fig. 2c). As expected, Gag was not present in the supernatant from a control transfection where the Env-encoding plasmid is excluded (Fig. 2c, lane 2). This demonstrates that the amount of Gag in the supernatant could be used as a surrogate for released FVV virions. Thus, a similar number of virions were released by transfected cells, irrespective of the vector size. The independence of virion release on vector size was consistent between independent replicates (not shown). In contrast, small variations between samples, which were insufficient to account for the 100-fold differences in titre measured by RT-qPCR and Alu-qPCR, varied between replicates, indicating that these were due to normal biological and/or technical variation.

### Muscle derived cells are efficiently transduced by FVV

**Figure 3:**
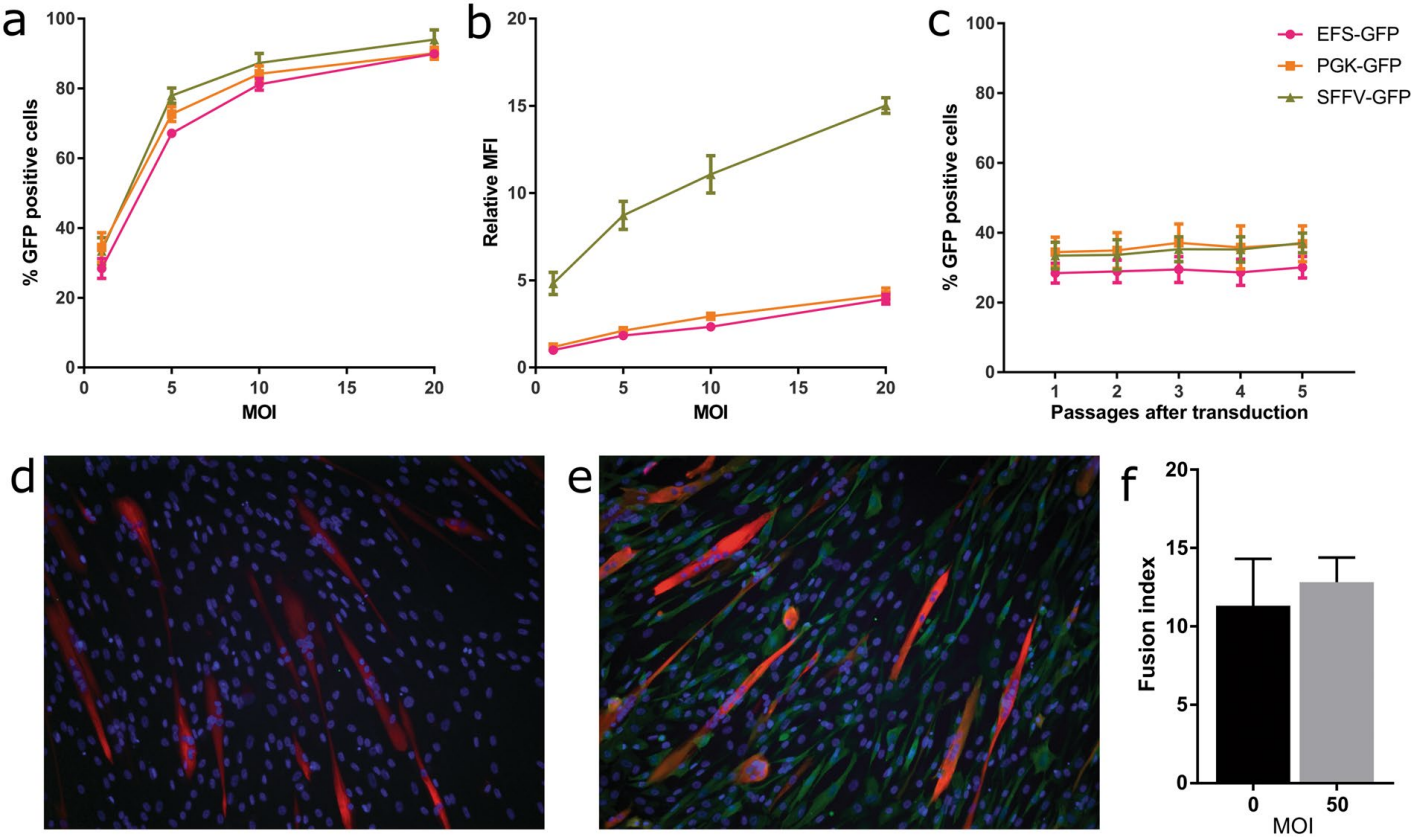
FVV transduction efficiency, promoter activity and toxicity in muscle derived cells. (**a**–**c**) FVVs carrying an EFS, PGK or SFFV promoter to drive constitutive GFP expression were added at various MOIs to muscle derived cells (cell line 1). The percent of cells expressing GFP (**a**) and their median fluorescence intensity (**b**) was determined by flow cytometry 1 passage post-transduction. (**c**) The percent of cells expressing GFP following each passage up to 5 post-transduction using an MOI of 1 was determined to analyse stability of expression. (**d**,**e**) Immunofluorescence staining of muscle derived cells (cell line 2) following culture in differentiation medium with antibodies targeting the myosin heavy chain (red). GFP is shown in green and DAPI-stained nuclei in blue. Panel d was untransduced, panel e was transduced at a MOI of 50 with DF-EFS-GFP-WPRE. The number of myotubes staining positive for myosin heavy chain were counted in 5 randomly selected fields-of-view to give the fusion index (**f**). Bars show mean + SD.

Given the ability of FVV to deliver transgenes of at least 12 kb at titres sufficient for typical *ex vivo* applications (~10^6^ TU/ml), the suitability of FVV for gene therapy of the DMD and BMD was evaluated further. To determine the multiplicity of infection (MOI) necessary for efficient transduction, the ability of FVVs to deliver and express GFP efficiently in muscle derived cells was investigated. In parallel, the strength and stability of FVV-mediated GFP-expression under the control of the EFS, PGK or spleen focus forming virus (SFFV) promoters was compared. All vectors were of an identical design (DDF-*promoter*-GFP-WPRE). Each vector was added at different MOIs to muscle derived cells then the percent of cells expressing GFP and their median fluorescence intensity (MFI) was determined by flow cytometry (Fig. 3a,b **)**. The stability of expression from each promoter was examined in cells transduced at an MOI of 1, the lowest tested, by determining the percentage of cells expressing GFP following each passage from 1 to 5 post-transduction (Fig. 3c **)**. The effect of FVV on the function of muscle derived cells was evaluated by transducing cells at a high MOI of 50 and assessing their ability to form myotubes *in vitro* and comparing that to untransduced cells (Fig. 3d). Myotubes were defined as cells staining positive for myosin heavy chain (MF20) and containing 3 or more nuclei. Myotube formation was quantified using the fusion index, determined as the percent of total nuclei that are within a myotube (Fig. 3f).

High transduction efficiency (~80–90% of cells expressing GFP) was achieved by all vectors using an MOI of 10 or 20 (Fig. 3a). The physiological promoters, EFS and PGK, had similar activities, while the viral SFFV promoter exhibited approximately 5-fold higher activity at all MOIs tested (Fig. 3b). At an MOI of 1, approximately 30% of cells expressed GFP. This was found to be stable for at least 5 passages for all promoters (Fig. 3c), indicating that the provirus is not subjected to silencing during expansion of the muscle derived cells. Importantly, the ability of muscle derived cells to form myotubes *in vitro* was not impaired by FVV transduction, even at an MOI higher than necessary for efficient transduction (Fig. 3d–f).

### Delivery of the full-length DMD ORF to muscle derived cells by FVV

To test whether a full-length dystrophin construct could be delivered and expressed by FVV in muscle derived cells, we initially transduced them at an MOI of 10 with DDF-PGK-Dys (Table 1) and a new construct, DDF-PGK-Dys-oPRE which included an optimised WPRE (oPRE) (total insert size of 12 179 bp). The PGK promoter was chosen at this stage because of its favourable performance in genotoxicity assays^28^. However, no dystrophin expression was detected by immunofluorescence or Western blot analyses following transduction (not shown).

**Figure 4:**
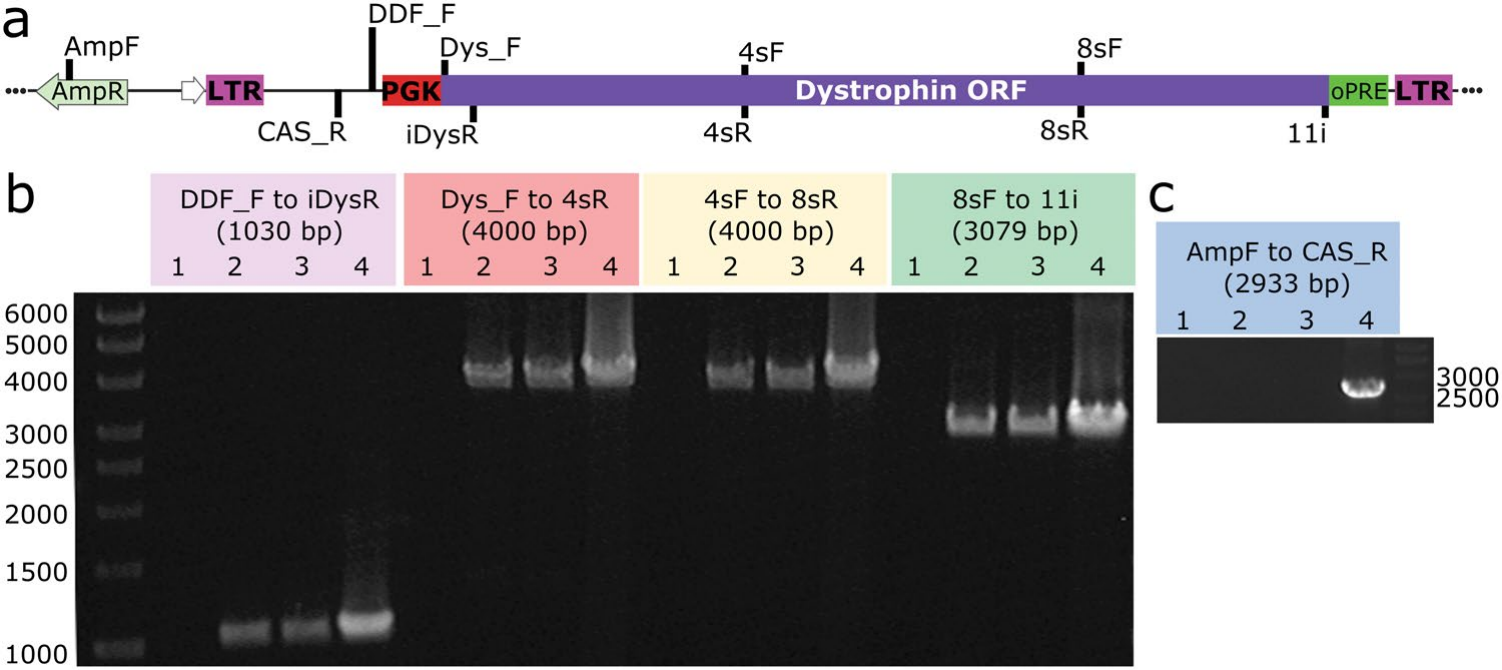
Full-length FVV is integrated in transduced muscle derived cells. (**a**) A map of the transfer plasmid pDDF-PGK-Dys-oPRE is shown (not to scale) with the positions of primers used for PCR in panels B and C indicated. PCR products were visualised following separation by DNA gel electrophoresis using the primers indicated above each lane (**b**,**c**). The expected amplicon size is given in brackets for each primer set. All lanes are numbered according to the PCR template. Template for lanes 1–3 were genomic DNA from muscle derived cells (cell line 2) where 1 was untransduced; 2 was transduced with FVV-PGK-Dys and 3 was transduced with FVV-PGK-Dys-oPRE. Lane 4 template was plasmid DNA (pDDF-PGK-Dys-oPRE). Numbers next to the DNA ladders show the size of the adjacent band in bp. Full length gel images are shown in Fig. S6b,c.

Titrating vectors by quantification of nucleic acids only requires the presence of the primer annealing sites which, in this study, target the LTR. Since delivery of truncated FVV could explain the lack of dystrophin expression, a series of PCRs were designed to span 12 kb of the provirus (from upstream of the promoter to the 3′ terminus of the dystrophin ORF) to determine whether the encoded vector was delivered in full (Fig. 4a,b). Due to its size and being split over 79 exons, the endogenous dystrophin gene could not provide template for these PCRs. However, if present, transfer plasmid carried over from vector production could have been used as template in the absence of genomic copies of provirus. To test for the presence of transfer plasmid in genomic DNA preparations, an additional PCR designed to amplify a region spanning the plasmid backbone and core vector sequences was performed (Fig. 4c).

As shown in Fig. 4b, the major PCR product from all transduced genomic DNA templates was of the expected size for full-length provirus and consistent with that of transfer plasmid template. As expected, no amplification of FVV-DNA occurred using genomic DNA from untransduced cells. Furthermore, transfer plasmid was not detected in genomic DNA samples (Fig. 4c), demonstrating that FVV, rather than carry-over transfer plasmid, provided the PCR template.

### Transduced muscle derived cells express dystrophin and can form myotubes

Having demonstrated that FVV was able to deliver the full-length dystrophin ORF to muscle derived cells, but dystrophin expression was undetectable using our assays, we optimised vector design to improve expression. The complete dystrophin ORF was codon-optimised for expression in human cells. Both the original full-length dystrophin sequence and the codon-optimised dystrophin ORF (coDys) sequence was inserted into the DDF transfer plasmid under the control of the EFS, PGK or SFFV promoters. For some constructs, the oPRE (596 bp) was added to potentially increase expression. In transfected 293 T cells, all transfer plasmids induced full-length dystrophin expression, as determined by Western blot analysis (Fig. S2). Putative dystrophin degradation products with lower molecular weights were also seen in many samples (Fig. S2). Equal volumes of unconcentrated vector from the transfected 293 T cells was applied directly to HT1080 cells. After one passage post-transduction, cells were examined for dystrophin expression by immunofluorescence microscopy. No dystrophin expression was detected in cells transduced with FVV encoding the non-optimised dystrophin ORF under the control of any promoter tested. In contrast, dystrophin-specific staining was detected in cells transduced by all FVVs encoding coDys (Table 2 and Fig. S3). Where tested, inclusion of the oPRE downstream of coDys increased dystrophin expression. The MOI was retrospectively determined by qPCR and ranged between 0.01 and 0.06 for all vectors. Optimisation of the dystrophin ORF did not affect FVV titre (Table 2).

**Table 2:** Immunofluorescence analysis of dystrophin expression in FVV transduced HT1080 cells.

FVV (DDF) insert	Signal intensity^a^	MOI^b^
PGK-Dys	−	0.01
PGK-Dys-oPRE	−	0.01
SFFV-Dys	−	0.06
SFFV-Dys-oPRE	−	0.03
EFS-coDys	+	0.04
EFS-coDys-oPRE	+++	0.03
PGK-coDys-oPRE	+++	0.02
SFFV-coDys	++	0.03
SFFV-coDys-oPRE	++++	0.02

^a^The relative dystrophin staining intensity was subjectively scored between – (no specific staining) and ++++ (strongest specific staining). Representative images are shown in Fig. S3. ^b^Genomic DNA from the transduced cells was used to retrospectively determine the MOI.

**Figure 5:**
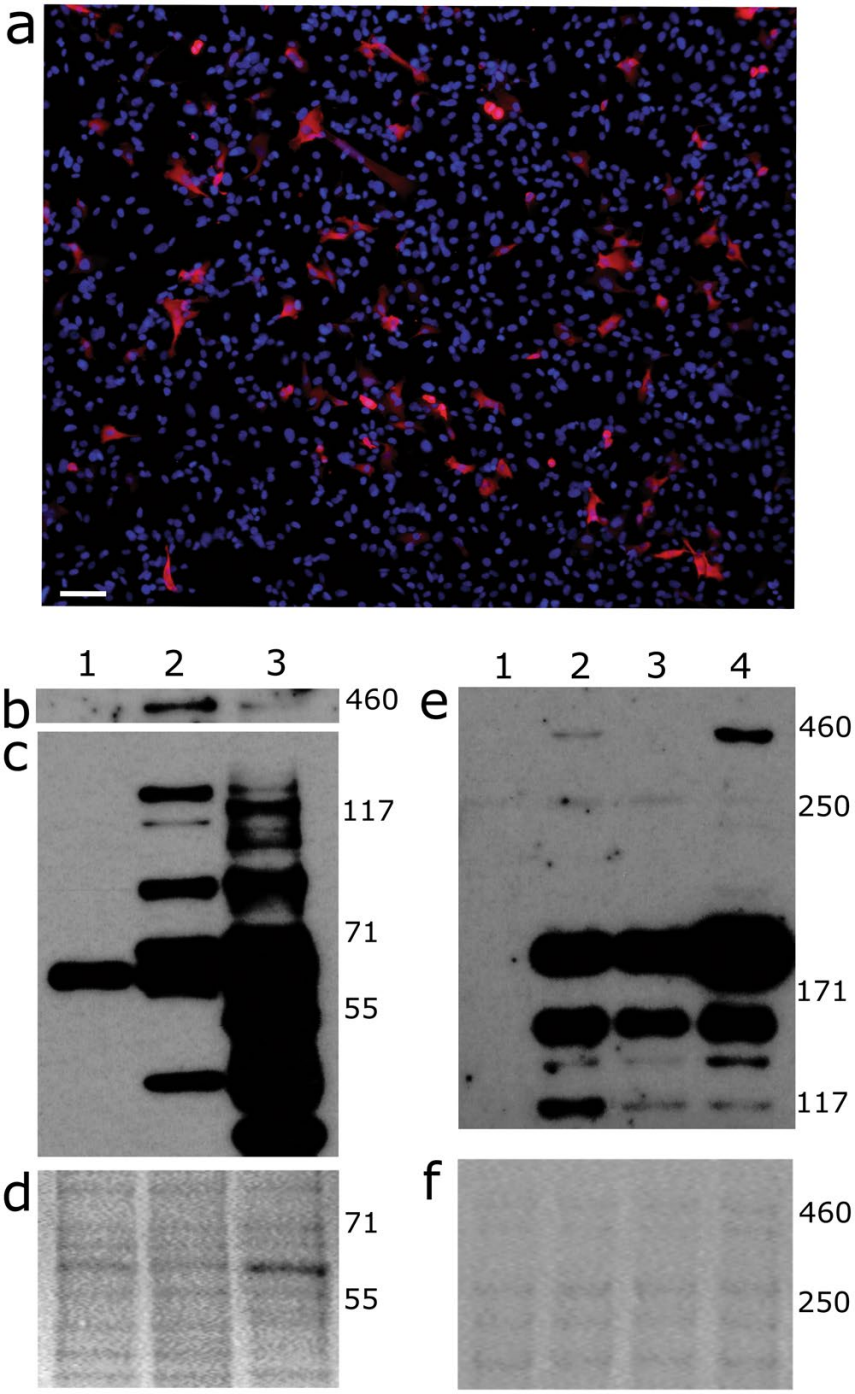
Full-length dystrophin expression in transduced HT1080 cells. Vectors DDF-PGK-coDys-oPRE and DDF-SFFV-coDys-oPRE were applied to HT1080 cells at an MOI of 2. (**a**) Cells transduced with DDF-SFFV-coDys-oPRE were analysed for dystrophin expression (red) by immunofluorescence. Nuclei were stained with DAPI (blue). Scale bar = 50 µm. (**b**–**d**) HT1080 cell lysates were analysed by Western blot for dystrophin expression. Lanes: 1, untransduced; 2, transduced with DDF-PGK-coDys-oPRE; 3, transduced with DDF-SFFV-coDys-oPRE. (**b**) Specific signal was detected near the 460 kDa marker following a long exposure period. (**c**) Lower molecular weight bands were detected following shorter exposure time. Full-length blot shown in Fig. S6d. (**d**) Ponceau S staining served as a loading control. (**e**) Lysates from consecutive passages of the DDF-PGK-coDys-oPRE transduced cells were analysed by Western blot for dystrophin expression. Lanes: 1, untransduced; 2–4 are lysates taken from the same transduced HT1080 cells after 1, 2 and 3 passages post-transduction, respectively. Full-length blot shown in Fig. S6e. (**f**) Ponceau S stain served as a loading control.

Production of the most promising vectors; DDF-PGK-coDys-oPRE and DDF-SFFV-coDys-oPRE; was scaled up and vector concentrated by ultracentrifugation to allow transduction at a higher MOI. Concentrated vectors were applied at an MOI of 2 to HT1080 cells. Following 1 passage post-transduction, dystrophin expression was assessed by immunofluorescence and Western blot analysis. Immunofluorescence showed that dystrophin-specific staining was detected in between 10–15% of DDF-SFFV-coDys-oPRE transduced cells (Fig. 5a). In contrast, few DDF-PGK-coDys-oPRE transduced cells had dystrophin-specific staining (not shown). Despite this, full-length dystrophin expression (and putative degradation products) were observed by Western blot analysis on cell lysates from both transductions (Fig. 5b–d). The DDF-PGK-coDys-oPRE transduced cells were further used to assess the stability of dystrophin expression after cell expansion (Fig. 5e,f). Full-length dystrophin expression was detected at 1 and 3 passages post-transduction, supporting previous data showing that large vectors are both integrated and full-length (Figs 2b and 4b). The observed variation in expression levels between passages may be expected since samples could be not collected in parallel (serial passages of the same sample). This was not investigated further. Rather, since both vectors demonstrated an ability to express full-length dystrophin, we tested these vectors in human muscle derived cells.

**Figure 6:**
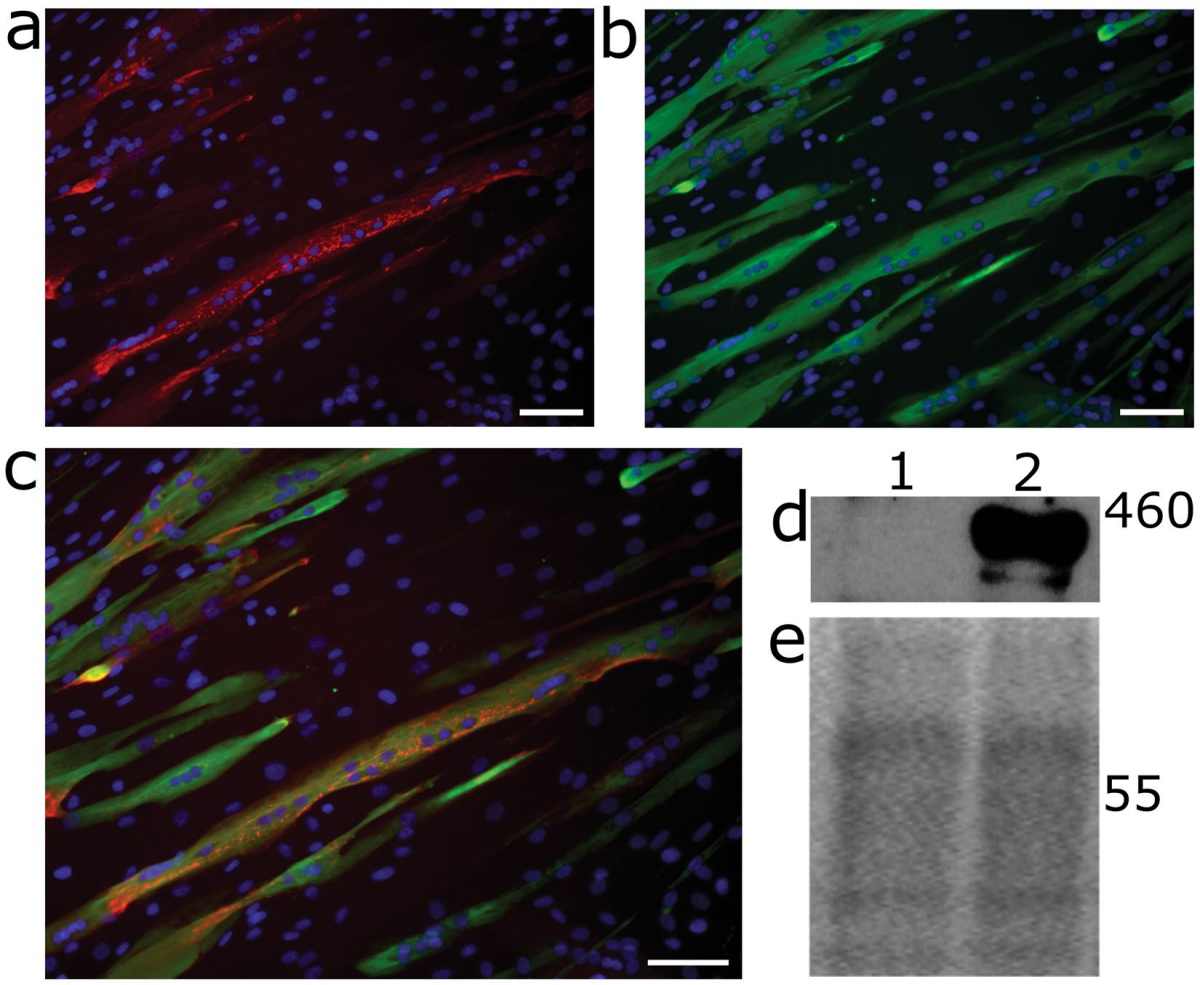
Transduced muscle derived cells form myotubes *in vitro* and express full-length dystrophin. (**a**–**c**) Representative immunofluorescence photomicrographs of muscle derived cells (cell line 3) transduced at an MOI of 1 with DDF-SFFV-coDys-oPRE showing expression of dystrophin (**a**) and myosin heavy chain (**b**) with DAPI stained nuclei (blue) following differentiation. A merge of a and b is shown (**c**). Scale bar is 25 µm. (**d**) Western blot on lysates from muscle derived cells (cell line 2). Lane 1 - untransduced; lane 2 - transduced with DDF-SFFV-coDys-oPRE at an MOI of 1. Full-length blot is shown in Fig. S6f. Ponceau S staining served as a loading control (**e**).

Both vectors were applied to muscle derived cells at an MOI of 10. However, this was detrimental to cell proliferation and transduction was inefficient when assessed by qPCR (Fig. S4). To limit toxicity, the muscle derived cells were transduced at a lower MOI using DDF-SFFV-coDys-oPRE. At a MOI of 1, cell proliferation was similar to untransduced cells (not shown). After 1 passage post-transduction, transduced cells were analysed for dystrophin expression and the ability to form myotubes *in vitro*. Coexpression of dystrophin and myosin heavy chain was observed by immunofluorescence, demonstrating that transduced cells could differentiate into myotubes *in vitro* (Fig. 6a–c). Furthermore, Western blot analysis confirmed robust full-length dystrophin expression (Fig. 6d).

## Discussion

In this study, we evaluated the effect of increasing FVV size on titre. Interestingly, incorporation of any dystrophin ORF sequence downstream of an EFS-GFP construct resulted in undetectable GFP-expression in transduced cells. This may indicate that the dystrophin ORF sequence is unstable, degraded or unable to be exported from the nucleus efficiently in a FVV context. Two PCR-based methods were used to determine the titre of different sized constructs at distinct points in the FVV life-cycle. Firstly, we measured levels of packaged FVV RNA (Fig. 2a), a measure which does not depend on successful reverse transcription and integration. Secondly, we measured integrated provirus in the genomic DNA of transduced cells using Alu-qPCR. This technique quantifies FVV that has successfully completed all stages of the vector life-cycle. Remarkably, there was a close correlation between relative titres for all vector sizes calculated by both methods. This indicates that a major contributor to the reduction of titre as vector sizes increase is at or prior to the RNA packaging step. This could be due to inefficient nuclear export of large vector genomes, instability of the longer vector RNAs and/or reduced packaging efficiency. Interestingly, the amount of capsid released by vector producing cells was similar for all vector sizes. Similar results have been reported for lentiviruses vectors^25^. This would indicate that, when the availability (or packaging efficiency) of vector genomes is limiting, excess virions are released without a vector genome. Genome-less virions, or virus-like particles, have been described previously for foamy viruses^29^ and other retroviruses^30^ where cellular RNAs substitute for viral RNA when it is absent.

While AAV vectors have a defined packaging limit of approximately 5 kb^24^, a distinct packaging limit was not found for lentivirus vectors^25^. Rather, similar to our findings, a semi-logarithmic reduction in titre was observed as vector size increased. However, whereas we report that a 100-fold reduction in titre occurs with a provirus size of approximately 15 kb (12.5 kb insert), lentivirus vectors suffered a 100-fold reduction in titre at approximately 9 kb (6 kb insert). At that size, FVVs have only 10-fold reduced titre. Since lentivirus and FVVs with small inserts can be produced to similar titres^31, 32^, it appears that FVVs have an advantage over LVVs of being able to deliver approximately twice as much cargo as LVVs at useful titres. This information will be valuable for approaches where the stable delivery of multiple and/or large transgene cassettes is required.

One target for which an efficient means of delivering vector encoding a large transgene is desired is that of DMD. With an ORF of ~11 kb, both AAV and lentivirus vectors are unable to deliver it efficiently in its entirety. Using FVV, we describe the first integrating vector able to do so. In human muscle derived cells transduced with FVV encoding full-length dystrophin, the whole promoter-dystrophin transgene cassette could be amplified by PCR, showing that vector was delivered in full (Fig. 4). Following expression optimisation, full-length dystrophin was readily detected in transduced muscle derived cells (Fig. 6d). Consistent with data in (Fig. 2), the median titre of FVV containing a 12 kb insert (promoter-coDys-oPRE) was approximately 200-fold lower than the median titre of FVVs with a 1–2 kb insert with median titres of 1.3 × 10^6^ TU/ml and 2.4 × 10^8^ TU/ml, respectively (Fig. S5). This would be sufficient for efficient transduction of muscle derived cells based on typical seeding densities (10^4^ cells per cm^2^) and volume constraints of cell-culture vessels. Accordingly, we applied four separate coDys-encoding FVV preparations to muscle derived cells at high MOI (10) during this study.

When muscle derived cells were transduced with FVV encoding GFP at a MOI of 50, cytotoxicity was not observed. In contrast, when FVV encoding coDys was applied at a MOI of 10, cell proliferation was reduced indicating cytotoxicity. The presence of 5% DMSO in our vector preparations did not cause this, since cells transduced with an equal volume of GFP-encoding FVV were unaffected. Contaminants, such as cellular debris and transfection reagent, are likely to have been co-concentrated with FVV. Scalable methods to concentrate and purify FVV by chromatography have been described^31^ that may alleviate toxicity due to such contaminants. However, at equal MOIs, FVVs encoding coDys are likely to contain approximately 200-fold more virions (mostly genome-less) than small GFP-encoding FVVs since only the functional titre, not the virion number, is affected by vector size (Fig. 2). Hence, by virion number, an MOI of 10 for coDys-encoding FVV is equivalent to an MOI of ~2000 using a GFP-encoding vector. Cytotoxicity from an MOI of 2000 would not be surprising in any cell type. Adjustments to the transfection ratio to balance particle assembly with vector genome availability could reduce the number of virus-like particles in a vector preparation and may reduce cytotoxicity at high MOIs. Such improvements may permit high transduction efficiency of coDys-encoding FVVs in muscle derived cells.

Given that FVV efficiently integrates a copy of its genome into the target cells DNA, the transgene is retained during cell expansion. Muscle progenitor cells cultured and expanded *ex vivo* can graft into injured muscle and contribute to muscle regeneration following local administration^33, 34^. Hence, FVV-mediated full-length dystrophin expression in muscle derived cells may be a good candidate for *ex vivo* gene therapy for DMD and BMD. While FVVs are also suitable for direct *in vivo* gene therapy^35^, efficient gene delivery is dependent on cell division^17^. Efficacy in muscle tissue, which is mostly non-dividing, would likely require FVVs to be pseudotyped with a muscle-progenitor-cell-specific envelope. Such an envelope has not yet been developed.

In conclusion, we have shown that FVV genome size negatively affects titre in a semi-logarithmic manner. Although similar to work described for lentiviruses, far longer transgene cassettes (approximately 12 kb) can be accommodated by FVV before titres are over 100-fold lower than normal. Uniquely, this enables FVV to package and deliver vector encoding the full-length dystrophin protein at titres exceeding 10^6^ TU/ml. To demonstrate this potential, we successfully expressed full-length dystrophin in human muscle derived cells. Improvements to vector manufacturing will enable pre-clinical testing of this vector for the treatment of DMD and BMD. This work highlights a valuable tool for stable delivery of large transgene cassettes and represents a milestone in the quest for a vector with potential to be developed into a cure for DMD.

## Materials and Methods

### Plasmid construction

All plasmids used in this study are listed in Table S1 with their source or details of their construction. Primers are given in Table S2. DNA was amplified by PCR using Q5 Hot Start High-Fidelity 2x Master mix and digested, ligated or assembled using restriction enzymes, T4 DNA ligase or Gibson Assembly Master Mix as appropriate (all from NEB, Hitchin, UK). Plasmids were propagated in NEB 10-Beta *E. coli* (NEB) and extracted for transfection using Qiagen Plasmid *Plus* kits.

### Cell culture

Human cells were obtained from the MRC Centre for Neuromuscular Diseases Biobank. All patients or their legal guardians gave written informed consent. All methods were carried out in accordance with relevant guidelines and regulations. All experimental protocols were approved by the NHS research ethics service, Hammersmith and Queen Charlotte’s and Chelsea Research Ethics Committee: Setting up of a Rare Diseases biological samples bank (Biobank) for research to facilitate pharmacological, gene and cell therapy trials in neuromuscular disorders (REC reference number 06/Q0406/33) and the use of cells as a model system to study pathogenesis and therapeutic strategies for Neuromuscular Disorders (REC reference 13/LO/1826), in compliance with national guidelines regarding the use of biopsy tissue for research.

Culture of 293 T and HT1080 cells followed standard methods, described previously^12^. Three separate muscle stem cell lines, each derived from different male DMD patients, were used in this study. This is indicated in the Figure legends as follows: Cell line 1 - skeletal muscle progenitor cells isolated from a 2-year-old patient (purchased from DVbiologics, Costa Mesa, CA); Cell line 2 - pD2 cells, previously described^36^, which are pericytes isolated from the extensor digitorum brevis muscle of an 11-year-old patient with a deletion of dystrophin exons 45–50; Cell line 3 - myoblasts isolated from quadriceps of a 3-year old patient with a mutation in dystrophin exon 42.

Cell line 1 was maintained on rat tail collagen (Fisher Scientific, Loughborough, UK) coated surfaces in Muscle Cellutions Medium (DVbiologics) supplemented with 10 ng/ml basic fibroblast growth factor (Fisher Scientific), as recommended by the cell supplier. Cell lines 2 and 3 were maintained in M10 medium on collagen coated plates at 37 °C, 5% O_2_, 5% CO_2_. M10 medium was comprised of Megacell Dulbecco’s Modified Eagle’s Medium, 2 µM glutamine, 1% non-essential amino acids, 0.1 mM β- mercaptoethanol (all from Sigma-Aldrich, Dorset, UK), 5 ng/ml basic fibroblast growth factor (Peprotech, London, UK) and 10% fetal bovine serum (Fisher Scientific, Loughborough, UK). For myogenic differentiation, cells were grown until confluent on Matrigel (BD biosciences) coated plates, then the medium was replaced by Megacell Dulbecco’s Modified Eagle’s Medium containing 2% fetal bovine serum and cells cultured for 7 days at 37 °C, 5% CO_2_.

### FVV production, titration and transduction

The production and concentration of FVV and its titration by flow cytometry was performed as previously described^12^. The Alu-qPCR was performed according to a published protocol^37^, except that primers in the first round PCR were Alu-1, Alu-2 and 203_R, followed by 203_F, 203_R and 203_P in the second round qPCR. Primer sequences are given in Table S2. For RT-qPCR, RNA was extracted from 10 µl of concentrated vector using the Qiagen QIAamp viral RNA mini kit (Qiagen, UK). Co-purified DNA was removed using the Turbo-free DNA kit (Fisher Scientific). FVV-specific RNA was quantified using the Qiagen OneStep RT-PCR kit (Qiagen) with primers 203_F, 203_R and 203_P. For transduction, HT1080 fibroblasts or muscle derived cells were seeded at 10^4^ cells per cm^2^. Between 6–20 hours later, vector was applied and transduction was enhanced by ‘spinoculation’ by centrifuging cells at 1200 g at 30 °C for 90 minutes.

### Amplification of vector provirus from genomic DNA of transduced cells by PCR

Cells were lysed in 50 mM Tris-HCl, pH 8, 200 mM NaCl, 20 mM EDTA, 1% SDS and 40 µg proteinase K at 56 °C overnight. Genomic DNA was isolated by organic extraction. PCR reactions were carried out using Q5 Hot Start High-Fidelity 2x Master mix containing 100 ng of genomic DNA or 0.1 ng of plasmid DNA. The primers used are shown in Fig. 4 and their sequences are given in Table S2.

### Western blot analysis

Cells were lysed in RIPA lysis buffer (Thermo Fisher) protein concentrations determined using the DC protein assay (Bio-Rad, UK). Equal amounts of protein were separated in a 3–8% NuPAGE Tris-Acetate gel (Thermo Fisher), according to the manufacturer’s recommendation and transferred to 0.45 μm pore polyvinylidene fluoride membranes. Ponceau S staining constituted a loading control, as described by others^38^. Rabbit-anti-dystrophin (ab15277 from Abcam, Cambridge, UK) was used at 0.2 µg/ml. A human anti-PFV serum was diluted 1 in 5000 for use. Appropriate horse-radish-peroxidase conjugated antibodies and chemifluorescence reagents were used for detection.

### Immunofluorescence analysis

Cells were cultured on Lab-Tek Permanox Chamber Slides (Sigma-Aldrich) or glass coverslips and fixed in 10% formalin. Rabbit-anti-dystrophin antibody (ab15277 from Abcam) was used at 0.4 µg/ml. Mouse-anti-MF20 antibody, deposited to the DSHB by Fischman, D.A. (DSHB Hybridoma Product MF 20), targets the myosin heavy chain and diluted 1 in 100 for use. Alexafluor-488 or -594 conjugated secondary antibodies were applied at 6.7 µg/ml. Images were captured with a Leica DM 4000B microscope using Metamorph software or a Nikon Eclipse TE2000S microscopy using Nikon ACT-1 software. The fusion index was determined as the percent nuclei within MF20 positive myotubes (containing at least 3 nuclei) of the total nuclei in the field of view.

## Electronic supplementary material

Supplementary material
