# Supplementary material for: Delivery of large transgene cassettes by foamy virus vector
Source: Sci Rep. 2017 Aug 14;7:8085. doi: 10.1038/s41598-017-08312-3 (PMC5556010; doi:10.1038/s41598-017-08312-3)
Supplement: Supplementary file 1 — Supplementary material [file 41598_2017_8312_MOESM1_ESM.pdf]

Delivery of large transgene cassettes by foamy virus vector.

Nathan Paul Sweeney, Jinhong Meng, Hayley Patterson, Jennifer E. Morgan, Myra McClure.

Supplementary Material

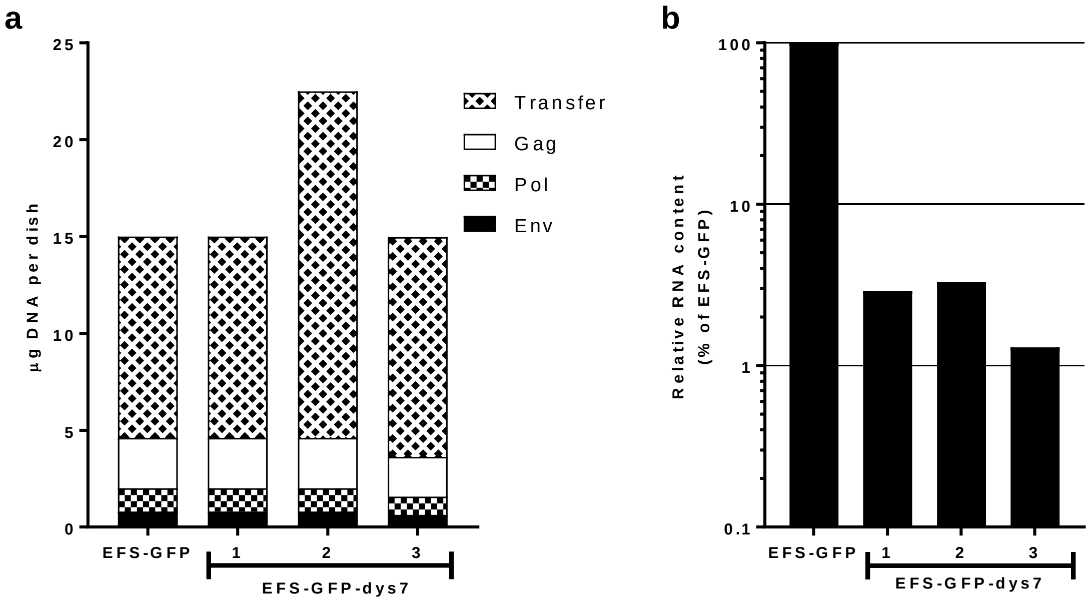

Figure S1 – Maintaining mass or molar plasmid ratios for large transfer vector production does not affect titre. (a) The amount of each plasmid (per 55 cm^2 dish) co-transfected in 293T cells for production of FVV is shown for DF-EFS-GFP and transfections 1-3 for DF-EFS-GFP-dys7. Transfection 1 uses the same mass of each plasmid as for EFS-GFP, whereas transfections 2 and 3 retain the same molar ratio of each plasmid by increasing the mass of transfer vector added (transfection 2) or decreasing the mass of packaging plasmids added (transfection 3). (b) The relative quantity of FVV RNA released into the cell-culture supernatant from the various transfections was quantified by RT-qPCR.

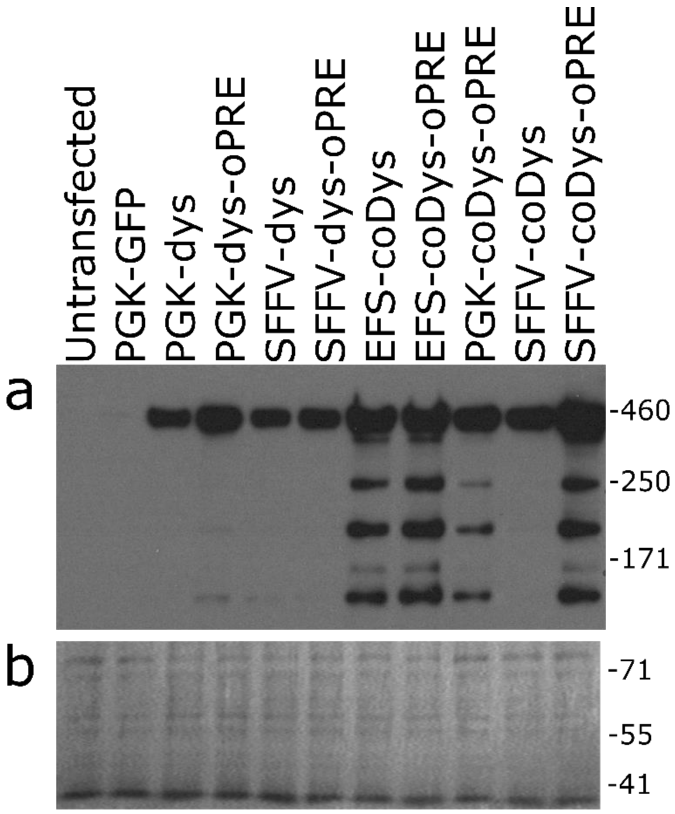

Figure S2 – Full-length dystrophin is expressed from transfer plasmids in transfected 293T cells. (a) Cell lysates from 293T cells transfected with FVV transfer plasmid pDDF, carrying the insert labelled above each lane, were analysed by Western blotting for dystrophin expression. (b) Ponceau S staining was used as a loading control.

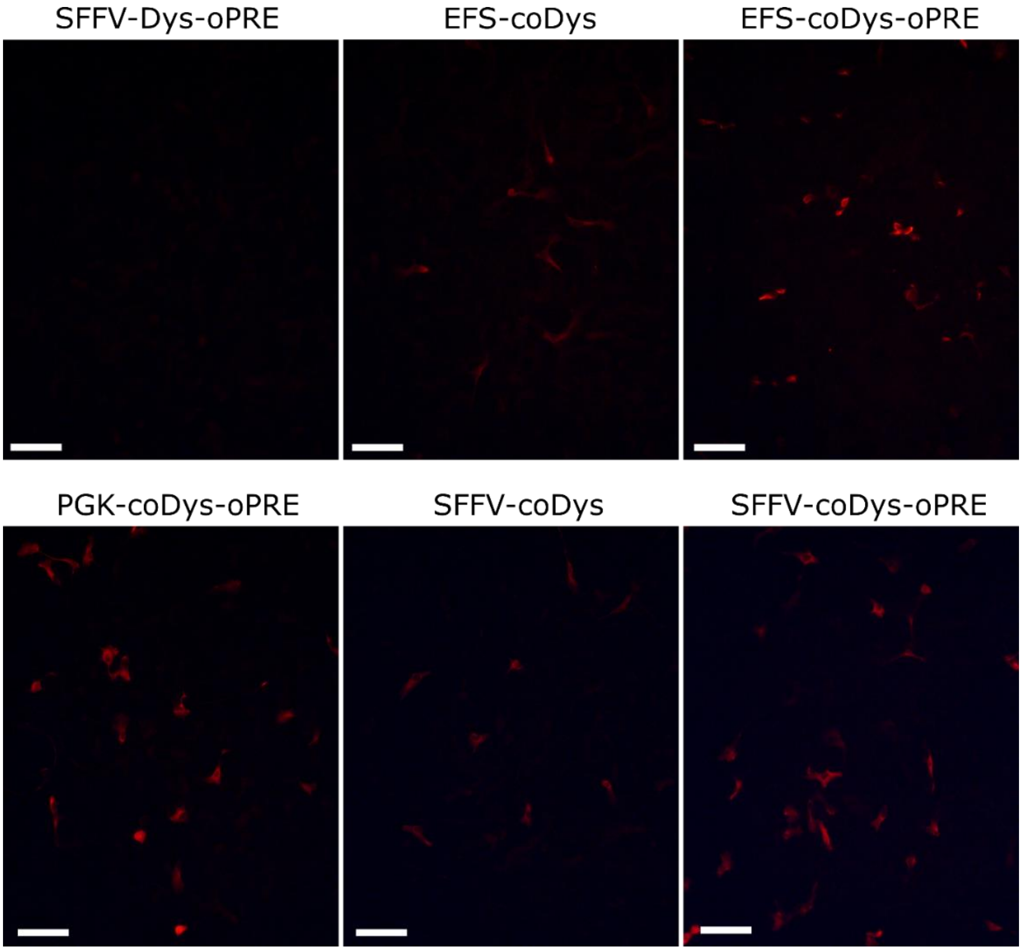

Figure S3 – Immunofluorescence analysis of dystrophin expression in FVV transduced HT1080 cells. Photomicrographs of transduced HT1080 cells are shown following immunostaining to detect dystrophin expression. Cells were confluent in all images and acquisition settings were the same for each sample. Where available, fields of view containing a high number of dystrophin-expressing cells were selected. The transgene cassette within each FVV (DDF) used to transduce each sample is labelled above each photomicrograph. Of the transductions that did not result in detectable dystrophin expression, only DDF-SFFV-Dys-oPRE is shown. Results are summarised in Table 2. Scale bar = 20 µm.

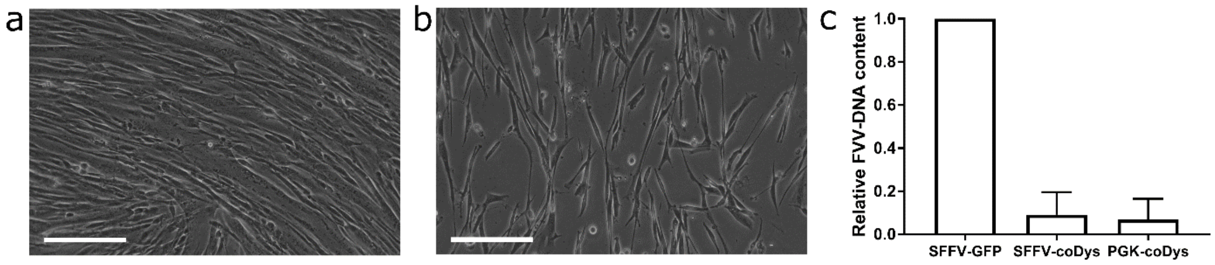

<u>Figure S4 – Transduction of muscle stem cells with coDys encoding FVV at high MOI affects proliferation and is ineffective.</u> Muscle stem cells (lines 2 and 3) were transduced, in parallel, at a MOI of 10 with DDF-SFFV-coDys-oPRE and DDF-PGK-coDys-oPRE. Representative photomicrographs show muscle stem cells (line 3) that were mock-transduced (a) or transduced with DDF-SFFV-coDys-oPRE at an MOI of 10 (b) taken 4 days post-transduction. The amount of FVV-DNA in genomic DNA preps (data pooled for lines 2 and 3) taken 2 passages post-transduction with the FVVs indicated (DDF-*promoter-transgene*-oPRE) was determined by qPCR (c). Mean + SD is shown.

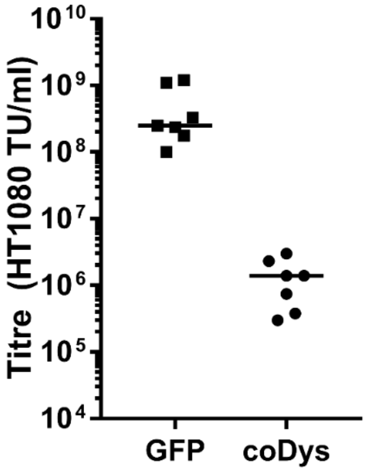

Figure S5 – Titres of concentrated FVVs produced during this study. Where determined, the titre of all small (<2 kb insert) GFP-encoding vectors or full-length coDys-(>12 kb insert) encoding vectors produced during the course of this study (containing any promoter) is shown. Each point represents a different vector preparation. The horizontal line shows the median average.

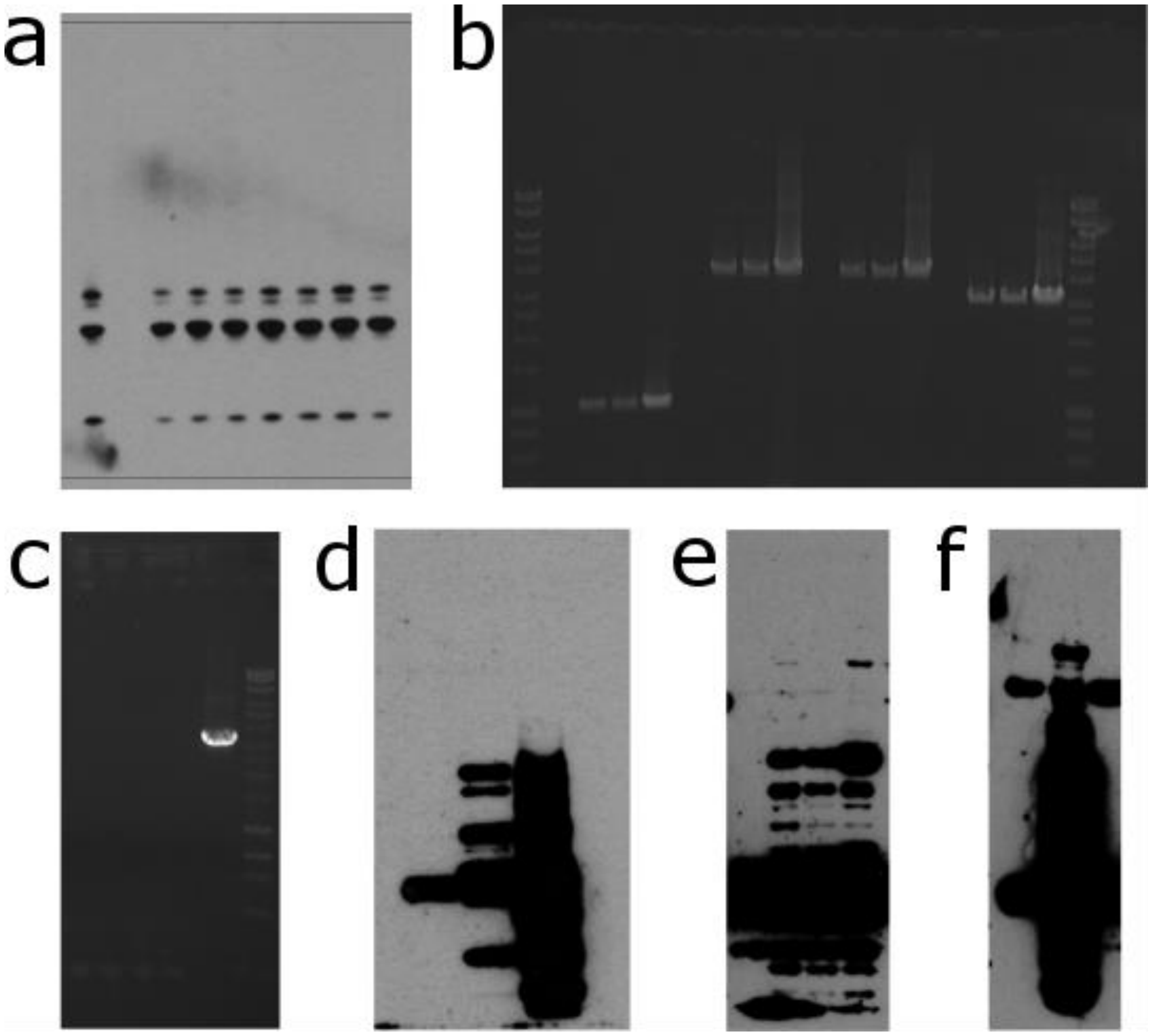

<u>Figure S6 – Full-length DNA gels and Western blots</u>

Full-length images of cropped Western blots and DNA gels from the main text are shown. (a) is full-length blot of Fig. 2c and has blot top and bottom edges marked for visibility; (b) is full-length gel of Fig. 4b; (c) is full-length gel of Fig. 4c; (d) is full-length blot of Fig. 5c; (e) is full-length blot of Fig. 5d; (f) is full-length blot of Fig. 6d.

Table S1 – Plasmids used in this study

Name	Description and source or construction
pcoSE	Encodes SFV_{mac} Env [1], provided by D. Lindemann (Dresden, Germany)
pcoPG4	Encodes PFV Gag [2], provided by D. Lindemann (Dresden, Germany)
pcoPP wt	Encodes PFV Pol [2], provided by D. Lindemann (Dresden, Germany)
pCIhDysGFP	Encodes a Dystrophin.GFP fusion - Provided by Olivier Danos
pDF	Empty PFV transfer vector, provided by D. Russell (Seattle, WA) [3]
pDF-EFS-GFP	Previously described transfer vector [1]
pDF-EFS-GFP-WPRE	Previously described transfer vector [1]
pDF-EFS-ARSA-oPRE	Previously described transfer vector [1] contains the oPRE [4]
pDF-PGK-GFP	The murine PGK promoter was amplified using primers PGK_F and PGK_R and replaced EFS as a ClaI-KpnI fragment in pDF-EFS-GFP.
pDF-PGK-GFP-WPRE	The PGK promoter was transferred as a ClaI-KpnI fragment from pDF-PGK-GFP to replace EFS in pDF-EFS-GFP-WPRE.
pDF-EFS-GFP-dys7	pDF-EFS-GFP was amplified using primers Gib-Dys-eg and gib-dys7v. Dys7 was amplified from pCIhDysGFP using gib-eg-dysF and gib-dys7i. The fragments were joined by Gibson assembly.
pDF-EFS-dys8	As above, but using gib-dys8v and gib-dys8i.
pDF-EFS-dys9	As above, but using gib-dys9v and gib-dys9i.
pDF-EFS-dys10	As above, but using gib-dys10v and gib-dys10i.
pDF-EFS-dysFL	As above, but using gib-dys11v and gib-dys11i.
pDF-PGK-Dys	pDF-PGK was amplified with primers Gib-PGK-v and Gib-dys11v. Dys was amplified from pCIhDysGFP using primers Gib3F and Gib-dys11i. PCR products were fused using Gibson assembly.
pDF-PGK-Dys.GFP	As for DF-PGK-Dys but using primer Gib3R-gfp.
pDDF-empty	pDF was amplified in 3 parts – 1. AmpsF and CasII_DDF_R; 2. cPPT_DDF_F_and AmpsR; 3. CASII_DDF_F and cPPT_DDF_R. The pieces were joined by Gibson assembly.
pDDF-EFS-GFP	EFS-GFP was transferred as a ClaI/NotI fragment from pDF-EFS-GFP to pDDF-empty
pDDF-EFS-GFP-dys(n)	All variations of EFS-GFP-dys(n) were excided from the pDF- plasmid using AgeI/NotI and inserted into pDDF-EFS-GFP.
pDDF-PGK-GFP-WPRE	PGK-GFP-WPRE was transferred as a ClaI/NotI fragment from pDF-PGK-GFP-WPRE to pDDF-empty.
pDDF-PGK-dys	PGK-GFP-WPRE was excised from pDDF-PGK-GFP-WPRE using AgeI/NotI and replaced with PGK-Dys from pDF-PGK-Dys
pDDF-PGK-dys.GFP	As above but using pDF-PGK-Dys.GFP for the insert.
pDDF-PGK-Dys-oPRE	DDF-PGK-Dys was amplified using AmpsF and Gib-Dys11i from pDDF-PGK-Dys. The oPRE was amplified with the 3' transfer vector using NotI-oPRE_F and AmpsR. The products were assembled by Gibson assembly.
pDDF-SFFV-GFP-WPRE	The SFFV promoter was amplified using primers SFFV_F and SFFV_R and inserted into pDDF-PGK-GFP-WPRE as a ClaI/KpnI fragment.
pDDF-SFFV-Dys	DDF-SFFV was amplified from pDDF-SFFV-GFP-WPRE using primers AmpsF and SFFV-dys_R. Part of dystrophin was amplified using primers SFFV-dys_F and dys4sR. The fragments were fused by PCR using primers AmpSF and dys4sR. The remaining dystrophin sequences and vector backbone was amplified using dys4sF and AmpsR from pDDF-PGK-dys and the fragments were joined by Gibson assembly.
pDDF-SFFV-Dys-oPRE	As for pDDF-SFFV-Dys except pDDF-PGK-dys-oPRE was used as template rather than pDDF-PGK-dys.
pcoDys.full	The whole dystrophin ORF from pCIhDysGFP was codon optimised using

	Geneart (Thermo Fisher). Sequence is available on request.
pDDF-PGK-coDys-oPRE	coDys from pcoDys.full was inserted into pDDF-PGK-dys-oPRE as a KpnI/NotI fragment.
pDDF-EFS-coDys	coDys from pcoDys.Full was inserted into pDDF-EFS-GFP as a KpnI/NotI fragment.
pDDF-EFS-coDys-oPRE	coDys-oPRE from pDDF-PGK-coDys-oPRE was inserted into pDDF-EFS-GFP as a KpnI/AscI fragment.
pDDF-SFFV-coDys	coDys from pcoDys.full was inserted into pDDF-SFFV-GFP-WPRE as a KpnI/NotI fragment.
pDDF-SFFV-coDys-oPRE	coDys from pcoDys.full was inserted into pDDF-SFFV-dys-oPRE as a KpnI/NotI fragment.

Table S2 - Primers used in this study

**Name**	**Sequence (modifications)**
203F	AGATTGTACGGGAGCTCTTCAC
203P	TACTCGCTGCGTCGAGAGTGTACGA (5’ FAM, 3’ BHQ1)
203R	CAGAAAGCATTGCAATCACC
Alb_F	GCTGTCATCTCTTGTGGGCTGT
Alb_P	CCTGTCATGCCCACACAAATCTCTCC (5’ CALfluor gold 540, 3’ BHQ1).
Alb_R	ACTCATGGGAGCTGCTGGTTC
Alu-1	TCCCAGCTACTGGGGAGGCTGAGG
Alu-2	GCCTCCCAAAGTGCTGGGATTACAG
AmpsF	GAGATCCAGTTCGATGTAACCCACTCGTGC
AmpsR	GCACGAGTGGGTTACATCGAACTGGATCTC
CasII_DDF_F	CCAAACAATAATTGACTATATTGGACCTTT
CasII_DDF_R	TATAGTCAATTATTGTTTGGGATATCCTTT
CPPT_DDF_F	CAACACAGGAGAGGGCCGGCCTCGACGGTATCGAT
CPPT_DDF_R	GCCGGCCCTCTCCTGTGTTGTTTAAAGCAAGTTGT
Df_Opre_R	GGCGCGCCTGATCTAGAGTCacgacaacaccacggaattatca
Gib(Dys)Sffv	Acttcttcccaccaaagcatggtacccgggcgactcagtc
Gib-(Sffv)Dysf	Gactgagtcgcccgggtaccatgctttggtgggaagaagt
Gib3f	cctcatctccgggcctttcgacctggtaccatgctttggtgggaagaagtagaggactgt
Gib3r	ACGGCGCGCCTGATCTAGAGTCGCGGCCgcttaCATTGTGTCctctctcattggctttccagg
Gib3R-GFP	ACGGCGCGCCTGATCTAGAGTCGCGGCCgcttacttgtacagctcgtccatgccgagagt
Gib-Dys10i	CTTGCGCGCCTGATCTAGAGTCgcggccgcaaagtgctttagactcctgtacctgaatcc
Gib-Dys10v	ggattcaggtacaggagtctaaagcactttgcggccgcGACTCTAGATCAGGCGCGCAAG
Gib-Dys11i	TCgcggccgcCATTGTGTCctctctcattg
Gib-Dys11v	gGACACAATGgcggccgcGACTCTAGATCA
Gib-Dys7i	CTTGCGCGCCTGATCTAGAGTCgcggccgcggtcttcaagcttttttcaagctgcccaa

Gib-Dys7v	ttgggcagcttgaaaaaaagcttgaagaccgcggccgcGACTCTAGATCAGGCGCGCAAG
Gib-Dys8i	CTTGCGCGCCTGATCTAGAGTCgcggccgcatattctctgttatcatgtggacttttctg
Gib-Dys8v	cagaaaagtccacatgataacagagaatatgcggccgcGACTCTAGATCAGGCGCGCAAG
Gib-Dys9i	CTTGCGCGCCTGATCTAGAGTCgcggccgcgcaaggtcattgacgtggctcacgttctct
Gib-Dys9v	agagaacgtgagccacgtcaatgaccttgcgcggccgcGACTCTAGATCAGGCGCGCAAG
Gib-Dys-Eg	acagtcctctacttcttcccaccaaagcatctagctactagctagtcgagatctgagtcc
Gib-Eg-Dysf	ggactcagatctcgactagctagtagctagatgctttggtgggaagaagtagaggactgt
Gib-Egv	ccaaagcatctagctactagctagtcgaga
Gib-Pgk-v	accaaagcatggtaccaggtcgaaaggccc
Noti-Opre_F	gcGGCCGCtatacaaaagttggagcatctta
Sffv_F	GCATCGATgccattttgcaaggcatggaa
Sffv_R	Gcggtacccgggcgactcagtctgt
SFFV-dys_F	gactgagtcgcccgggtaccatgctttggtgggaagaagt
SFFV-dys_R	acttcttcccaccaaagcatggtacccgggcgactcagtc
Dys4sF	gcatattggcacagaccctaacagatggcg
Dys4sR	cgccatctgttagggtctgtgccaatatgc

Supplementary References

1 Sweeney, N. P. *et al.* Rapid and Efficient Stable Gene Transfer to Mesenchymal Stromal Cells Using a Modified Foamy Virus Vector. *Mol Ther* **24**, 1227-1236, doi:10.1038/mt.2016.91 (2016).

2 Stirnnagel, K. *et al.* Analysis of Prototype Foamy Virus particle-host cell interaction with autofluorescent retroviral particles. *Retrovirology* **7**, 45 (2010).

3 Trobridge, G., Josephson, N., Vassilopoulos, G., Mac, J. & Russell, D. W. Improved foamy virus vectors with minimal viral sequences. *Mol Ther* **6**, 321-328 (2002).

4 Schambach, A. *et al.* Woodchuck hepatitis virus post-transcriptional regulatory element deleted from X protein and promoter sequences enhances retroviral vector titer and expression. *Gene Ther* **13**, 641-645 (2005).
